# The Small GTPases Rab27b Regulates Mitochondrial Fatty Acid Oxidative Metabolism of Cardiac Mesenchymal Stem Cells

**DOI:** 10.3389/fcell.2020.00209

**Published:** 2020-04-15

**Authors:** Yue Jin, Yan Shen, Xuan Su, Jingwen Cai, Yutao Liu, Neal L. Weintraub, Yaoliang Tang

**Affiliations:** ^1^Vascular Biology Center, Medical College of Georgia, Augusta University, Augusta, GA, United States; ^2^Cellular Biology and Anatomy, Medical College of Georgia, Augusta University, Augusta, GA, United States

**Keywords:** Rab27b, mitochondrial oxidative metabolism, cardiac mesenchymal stem cells, fatty acid oxidation, exosome

## Abstract

Cardiac mesenchymal stem cells (C-MSCs) are endogenous cardiac stromal cells that play a crucial role in maintaining normal cardiac function. Rab27b is a member of the small GTPase Rab family that controls membrane trafficking and the secretion of exosomes. However, its role in regulating energy metabolism in C-MSC is unclear. In this study, we analyzed mitochondrial oxidative phosphorylation by quantifying cellular oxygen consumption rate (OCR) and quantified the extracellular acidification rate (ECAR) in C-MSC with/without Rab27b knockdown. Knockdown of Rab27b increased glycolysis, but significantly reduced mitochondrial oxidative phosphorylation (OXPHOS) with loss of mitochondrial membrane potential in C-MSC. Furthermore, knockdown of Rab27b reduced H3k4me3 expression in C-MSC and selectively decreased the expression of the essential genes involved in β-oxidation, tricarboxylic acid cycle (TCA), and electron transport chain (ETC). Taken together, our findings highlight a novel role for Rab27b in maintaining fatty acid oxidation in C-MSCs.

## Introduction

Cardiovascular disease (CVD) is the number one cause of mortality and morbidity worldwide, and its prevalence will increase with the progressive aging of the general population (Smith et al., 2012). CVD is characterized by a spectrum of alterations in cardiac energy and substrate metabolism (van Bilsen et al., 2004; Bertero and Maack, 2018). The heart consumes large amounts of energy in the generation of ATP, which is mainly supplied by oxidative phosphorylation (OXPHOS) in mitochondria (Bertero and Maack, 2018). Under normal conditions, about 60–90% of ATP required for the continuous contractile activity of the heart is primarily produced by oxidation of fatty acids (FA), while the remainder is derived from the oxidation of pyruvate (van der Vusse et al., 1992). Fatty acid oxidation (FAO) is a complex biological process involving mitochondrial β-oxidation, tricarboxylic acid (TCA) cycle activity, and the electron transport chain (ETC) (Lopaschuk et al., 2010). Changes in fatty acid metabolism can lead to a variety of CVDs. For example, excessive intake of fatty acids and beta-oxidation in obesity and diabetes can impair heart function. Additionally, changes in fatty acid β-oxidation during and after ischemia and in nutrient-depleted hearts may also contribute to cardiac pathology, affecting cardiac systolic function and cardiac efficiency (Liu et al., 2002; Lopaschuk et al., 2010).

Cardiac mesenchymal stem cells (C-MSC) that reside in adult hearts express the cardiac transcription factor GATA-4 and mesenchymal stem cell markers, including CD105, CD140, and Sca-1 (Ruan et al., 2018a, b). These C-MSC have cardiac reparative properties via paracrine mechanisms mediated by angiogenic factors (Tang et al., 2004, 2005) and/or exosomes (Chen et al., 2013; Wang et al., 2015; Campbell et al., 2016). Exosomes secreted by C-MSC are nano-size bilayer membrane vesicles (around 30∼150 nm in diameter) that can be absorbed by surrounding tissues (Vestad et al., 2017). Thus, proteins, RNA, and lipids from exosomes of C-MSC can enter adjacent cells (such as cardiomyocytes) and regulate their cellular signaling. We have reported that C-MSC-derived exosomes could protect cardiomyocytes from acute myocardial ischemia/reperfusion injury (Chen et al., 2013). Furthermore, recent studies have suggested that exosomes that are secreted from adipocytes and other cells in adipose tissue influence whole-body glucose and lipid metabolism (Ying et al., 2017; Flaherty et al., 2019). However, little is known about the metabolism of C-MSC contained in adult hearts.

The small GTPase family member Rab27b controls membrane trafficking and microvesicle transport, particularly the secretion of exosomes (Fukuda, 2013). While Rab27b can control cell–cell communications mediated by exosomes, including metabolism regulation, little is known about the role of Rab27b in regulating self-cell metabolism in C-MSC.

In this study, we first evaluated the role of Rab27b in the metabolism of adult C-MSCs and found that knockdown of Rab27b inhibits mitochondrial fatty acid β-oxidation, TCA, and ETC by decreasing the expression of related genes, resulting in mitochondrial respiratory depression in C-MSC.

## Materials and Methods

### C-MSC Isolation and Culture

Cardiac mesenchymal stem cells were isolated from the hearts of male C57BL/6 mice (The Jackson Laboratory, Bar Harbor, ME, United States) of 2 to 3 months old via an established protocol with modification (Tang et al., 2007; Ju et al., 2018a, b; Ruan et al., 2018a, b). Briefly, in step 1, ventricular tissue was minced into approximately 1-mm^3^-sized pieces and digested using 0.1% collagenase IV and 1 U/ml dispase in DMEM/F-12 for 1 h at 37°C. Then, cardiac explants were collected and incubated on fibronectin/gelatin-coated plates (0.5 mg fibronectin in 100 ml 0.1% gelatin) in DMEM containing 10% fetal bovine serum, 100 U/ml penicillin G, and 100 μg/ml streptomycin. Cultured cardiac explants were maintained until the small round phase-bright cells migrated from the adherent explants and proliferated over a fibroblast layer. In step 2, Sca-1 + cells were enriched from the phase-bright cells through a mouse hematopoietic lineage-depletion cocktail kit (STEMCELL Technologies, Vancouver, Canada), followed by enrichment for Sca-1 + cells via magnetic-activated cell sorting (MACS) with Sca-1 magnetic beads (Miltenyi Biotec Inc., Auburn, CA, United States) according to the manufacturers’ protocols. The selected Sca-1 cells were cultured and maintained in complete DMEM medium containing 10% fetal bovine serum, 100 U/ml penicillin G, 100 μg/ml streptomycin, 200 mmol/L L-glutamine, 55 nmol/L β-mercaptoethanol, and 1% MEM non-essential amino acids. Animal treatment protocols were approved by, and conducted in accordance with, animal welfare regulations of the Institutional Animal Care and Use Committee of the Medical College of Georgia.

### Flow Cytometry

Cardiac mesenchymal stem cells were first blocked with 5% rat serum (Sigma) and stained, respectively, with conjugated antibodies, including anti-CD105-APC (BioLegend), anti-CD140b-PE (BioLegend), or isotype-matched control antibody (BD Biosciences). Flow cytometry analysis of cultured C-MSC was performed with a BD LSRII flow cytometer from Augusta University.

### Exosome Purification

Exosomes released by the C-MSC were purified as previously described (Wang et al., 2015; Tang et al., 2017; Ju et al., 2018b; Ruan et al., 2018a, b; Su et al., 2018, 2019a). Briefly, after 48 h of cell culture in exosome-free medium, the supernatant was harvested and centrifuged at 150 × *g* for 10 min to eliminate cells, followed by filtration via 0.22-μm filter to remove cell debris. The filtered supernatant was ultracentrifuged by an SW-28 Ti rotor (Beckman Coulter Instruments, United States) at 100,000 × *g* for 120 min at 4°C to pellet the exosomes. The exosome pellets were resuspended in 1 ml PBS.

### Zeta Analysis

We measured the exosome particle size and concentration with nanoparticle tracking analysis (NTA) using ZetaView PMX 110 (Particle Metrix, Meerbusch, Germany) and the corresponding software ZetaView as previously described (Helwa et al., 2017; Shah et al., 2018; Rashid et al., 2019). Isolated exosome samples were appropriately diluted using 1 × PBS buffer (Life Technologies, Carlsbad, CA, United States) to measure the particle size distribution and concentration. NTA measurement was recorded and analyzed. The ZetaView system was calibrated using 100-nm polystyrene particles. The temperature was maintained at approximately 23°C.

### Immunofluorescent Staining

For cell staining, C-MSCs plated on an 8-well chamber slide (Thermo Fisher Scientific, United States) were fixed with 4% paraformaldehyde, followed by the permeabilization with 1% Triton X-100^TM^. After blocking with 5% goat serum, cells were incubated with rabbit anti-GATA4 (1:100; Aviva System Biology), rabbit anti-Rab27a (1:500; Cell Signaling), or rabbit anti-Rab27b (1:100; Millipore) at 4°C overnight. Secondary antibody incubation with goat anti-rabbit Alexa Fluor 555-conjugated (1:400, Invitrogen) was performed the following day, after which slides were mounted using VECTASHIELD HardSet Mounting Medium with DAPI (Vector Laboratories, United States).

### Lentiviral Vectors and Transfection

Lentiviral plasmids encoding shRNA targeting Rab27b mRNA (clone ID, MSH036525-31-LVRU6GH, MSH036525-32-LVRU6GH, MSH036525-33-LVRU6GH, and MSH036525-34-LVRU6GH) were purchased from Gene Copoeia. Lentiviral particles were produced in HEK293FT cells by cotransfecting the LVRU6GH shRNA plasmids, together with helper plasmids including pMD2.G and psPAX2 using lipofectamine 3000 reagents (Invitrogen). Viral supernatant was collected after 48 h. The lentiviral vectors were purified by adding PEG6000 (8.5% final concentration) and NaCl (0.4 M final concentration) to the 0.45 μM syringe filtered supernatant as previously reported (Su et al., 2019b). When C-MSCs reached 80% confluence, the purified lentivirus was added into medium containing 8 μg/ml of polypropylene for transduction. After 3 days of infection with lentiviral cells, hygromycin B (100 μg/ml) was added for cell selection.

### Isolation and Quantification of Messenger RNA

Total RNA was extracted by RNAzol RT (Molecular Research Center) according to the manufacturer’s instructions. cDNA was synthesized from total RNA using the RevertAid First Strand cDNA Synthesis Kit (Thermo Scientific). Quantitative PCR was performed using a PowerUp SYBR Green Master Mix (Thermo Fisher) on a CFX96 Touch real-time PCR detection system (Bio-Rad Laboratories, United States). The amplification was performed at 50°C for 2 min, at 95°C for 2 min, followed by 50 cycles of 95°C for 15 s, and at 60°C for 1 min, using the primers listed in Table 1.

**TABLE 1:** Primer sequences.

Gene	Sequence (5′–3′)
β-Actin FWD	AGAGCATAGCCCTCGTAGAT
β-Actin REV	GCTGTGCTGTCCCTGTATG
GAPDH FWD	TGACAAGCTTCCCATTCTCG
GAPDH REV	CCCTTCATTGACCTCAACTACAT
Rab27a FWD	CAGGAGAGGTTTCGTAGCTTAAC
Rab27a REV	GGCTTATCCAGTTTCGGACAT
Rab27b FWD	GTCCAGCAGTGTCCCAAAG
Rab27b REV	ATGACACACAAGGAGCAGATG
Sirt1 FWD	GTTGGTGGCAACTCTGATAAATG
Sirt1 REV	GTCATAGGCTAGGTGGTGAATATG
Ppargc1b FWD	AGGTGTGAGGGAAGCATAGA
Ppargc1b REV	CAAAGCCTTCTGGACTGAGTT
Acox1 FWD	CCTTGGCCAATGCTCTCATTA
Acox1 REV	CGCAGCAGTATAAACTCTTCCC
Acox3 FWD	CCCTAGAGAAGCTACGAGAACT
Acox3 REV	CAGGCAGTTAATCAGCACTAGAA
Hadha FWD	CCATGTCGGCCTTCTCAAA
Hadha REV	AGTGAAGAAGAAAGCTCTCACAT
Hadhb FWD	AGACCATGGGCCACTCT
Hadhb REV	CTTCTTGGCCAGACTATGAGAAC
Idh3a FWD	GGCCATCCATCTATGAATCTGT
Idh3a REV	GTATTCTCCTTCCGTGTTCTCTC
Ogdh FWD	CATGTATCACCGCAGGATCAA
Ogdh REV	GGTCTTTCCCATCACGACAG
Sdhd FWD	GATGCCGACATCGTGGTAAT
Sdhd REV	GTTACCGACTACGTTCATGGG
Uqcrq FWD	CTTTGCTGAAATAGCTTGGGAAG
Uqcrq REV	GAACCTGGCGCGGATAC

### Mitochondrial Membrane Potential Assay

We use a mitochondrial membrane potential detection JC-1 kit^TM^ (BD MitoScreen) to measure deltapsim according to instructions. The fluorescence intensity was measured at green fluorescence for JC-1 monomer and red fluorescence for JC-1 aggregate under a fluorescent microscope (Evos FL, Thermo). The deltapsim is represented by the ratio of JC-1 (red/green) on picture.

### Western Blotting Assay

Western blotting was performed as described before (Ruan et al., 2018a). Briefly, proteins (normalized for concentration) were resolved on 10% SDS–polyacrylamide gels and transferred onto Odyssey^®^ nitrocellulose membranes (LI-COR Biosciences). The membranes were blocked with Odyssey blocking buffer (LI-COR Biosciences, Lincoln, NE, United States) and probed with rabbit anti-Rab27a (1:1000; Cell Signaling), rabbit anti-Rab27b (1:1000; Millipore), rabbit anti-Tri-methyl-Histone H3 (Lys4) (1:1000; Cell Signaling), mouse anti-TBP (1:1000, Proteintech), and mouse anti-β-actin (1:5000, Novus Biologicals) at 4°C overnight. After washing with 1 × TBST, the membrane was incubated for 1 h at room temperature with IRDye 680 goat anti-rabbit IgG and IRDye 800 goat anti-mouse IgG (1:10,000, LI-COR Biosciences). The probed blot was scanned using an Odyssey infrared imager (LI-COR Biosciences).

### Cell Metabolism Assays

The Mito Stress Test Kit (Agilent) was used to measure the oxygen consumption rate (OCR). The Glycolytic Rate Assay Kit (Agilent) was used for measuring the extracellular acidification rate (ECAR). On the day prior to experimentation, the sensor cartridge for XF analyzer was hydrated in a 37°C non-CO_2_ incubator and cells were seeded at the density of 10,000 cells/well into XF96 cell culture microplates (for measurement of OCR) or 30,000 cells/well into XF24 cell culture microplates (Seahorse Bioscience) (for measurement of OCR and ECAR) and allowed to adhere to the plate overnight. On the day of the Seahorse assay, the cell culture medium was replaced with Seahorse XF DMEM base medium, without phenol red, supplemented with 10 mM glucose, 2 mM L-glutamine, and 1 mM pyruvate pH 7.4 and placed in a 37°C CO_2_-free incubator for 1 h. For the measurement of OCR value, oligomycin, phenylhydrazone (FCCP), and rotenone/antimycin A (Rot/AA, inhibitors of mitochondrial ETC), respectively, were added according to the manufacturer’s instructions and protocols. To determine effects of wild-type C-MSC-derived exosomes on OCR of C-MSC^sh–Rab27b^, we seeded C-MSC^sh–NC^ and C-MSC^sh–Rab27b^ at 30,000 cells/well into XF24 cell culture microplates using culture medium with exosome-depleted FBS overnight, and prime cells with wild-type C-MSC-derived exosomes (25 μg/well) for 1 h before OCR assay. For Palmitate-BSA FAO experiments, etomoxir (40 μM/well, Millipore) was added at 15 min before OCR analysis. BSA or Palmitate-BSA (Agilent Technologies, United States) was added to the wells immediately prior to initiate XF assay.

For the detection of ECAR value, Rot/AA and 2-deoxy-D-glucose (2-DG, an inhibitor of glycolysis) were added according to the manufacturer’s instructions and protocols. Finally, ECAR were determined and analyzed on the Agilent’s Seahorse Bioscience XF24 Extracellular Flux Analyzer (Agilent Technologies, United States) according to the manufacturer’s instructions and protocols (Agilent Technologies, United States). We first evaluated ECAR of C-MSC in the absence of glucose following by addition of glucose in 5 and 10 mM to compare the dosage of glucose on ECAR. Then, we compared the dosage of rotenone at a titration of 0.5 μM and 1 μM on ECAR in C-MSC^sh–NC^ and C-MSC^sh–Rab27b^.

### Statistics

Results are presented as the mean ± standard deviation (SD). Differences between two groups were analyzed using unpaired Student’s *t*-test, and the differences between three or more groups were analyzed using one-way analysis of variance (ANOVA). A value of *p* < 0.05 was considered statistically significant. Statistical analyses were conducted with GraphPad Prism 8.0 software.

## Results

### Characterization of C-MSC

**FIGURE 1:**
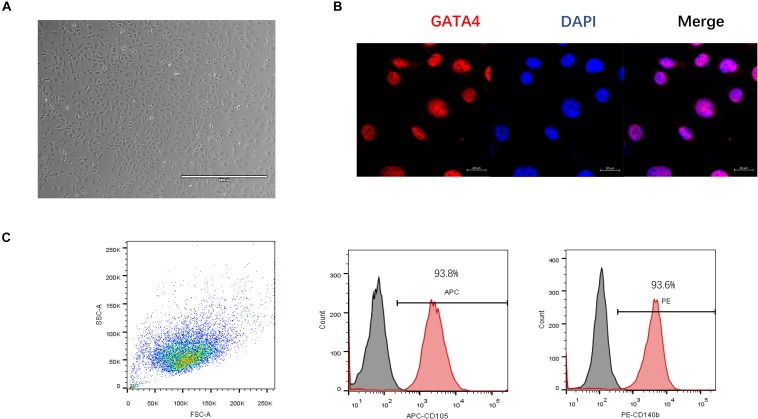
Phenotypic characterization of C-MSCs. **(A)** Cultured C-MSCs at passage 10, scale bar = 1000 μm. **(B)** Immunofluorescent staining of GATA4, a marker for early cardiac transcription factor (red); cell nuclei were counterstained with DAPI (blue) (scale bar = 20 μm). **(C)** Flow cytometric analyses of C-MSCs for the profile of the cell surface markers CD105 and CD140b.

Cardiac mesenchymal stem cells were obtained using a two-step procedure: cardiac-derived cells were grown from enzymatically digested minced adult mouse hearts and expanded, and then the C-MSCs were isolated using a hematopoietic lineage-depletion cocktail followed by enrichment for Sca-1 + cells via MACS sorting (Figure 1A). GATA4, an early cardiac transcription factor (Takeuchi and Bruneau, 2009), was positive in C-MSCs by immunofluorescent staining (Figure 1B). Surface marker expression was profiled by flow cytometry. Over 93.8% cells were positive for CD105, and 93.6% cells were positive for CD140b (Figure 1C). These data indicate that C-MSC represents a subpopulation of cardiac-derived mesenchymal cells (Nery et al., 2013).

### Lentiviral RNAi Vector-Mediated Knockdown of Rab27b in C-MSC

**FIGURE 2:**
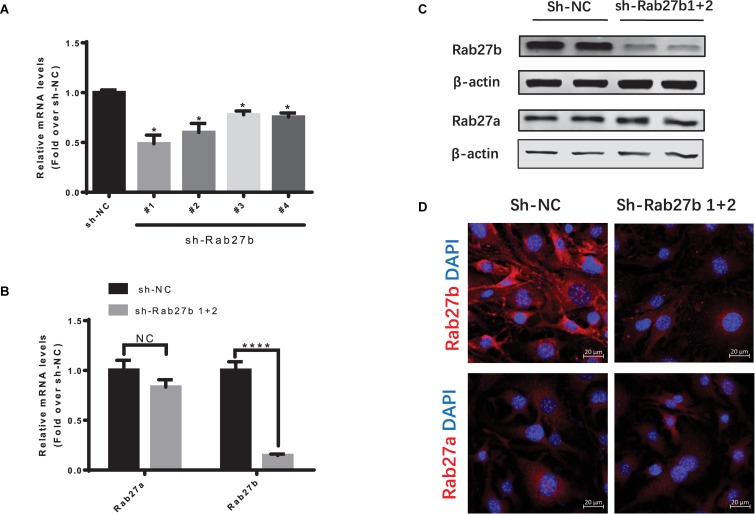
Knockdown of Rab27b in C-MSCs via lentiviral shRNAs vector transduction. **(A)** qRT-PCR analysis of Rab27b in C-MSCs cells infected with lentiviral vectors encoding shRNA targeting Rab27b mRNA (sh-Rab27b-1, sh-Rab27b-2, sh-Rab27b-3 and sh-Rab27b-4) or negative control (sh-NC). β-Actin was used as endogenous control. Results are shown as mean ± SD (*p < 0.05 vs. sh-NC, *n* = 3). **(B)** qRT-PCR analysis of Rab27a and Rab27b in C-MSCs infected with sh-Rab27b1 + 2 (lentiviral vectors encoding sh-Rab27b1 and lentiviral vectors encoding sh-Rab27b2) or negative control (sh-NC). GAPDH was used as endogenous control. Results are shown as mean ± SD (****p < 0.0001 vs. sh-NC, *n* = 3). **(C)** Western blotting of Rab27b and Rab27a protein using β-actin as a loading control. **(D)** Immunofluorescent staining of Rab27b and Rab27a (red) in C-MSC^sh–NC^ and C-MSC^sh–Rab27b1+2^; cell nuclei were counterstained with DAPI (blue) (scale bar = 20 μm).

To knock down the expression of Rab27b in C-MSCs, four lentiviral vectors with Rab27b small hairpin RNA (sh-Rab27b) were transfected into C-MSC, and a non-targeting shRNA (NC) was employed as control. The gene silencing efficiency of these shRNAs was evaluated by RT-PCR. As shown in Figure 2A, the lentiviral shRNA#1 and #2 efficiently downregulated the expression of Rab27b mRNA in C-MSCs. Thus, we analyzed C-MSCs that were stably double-transduced with lentiviral vectors encoding both sh-Rab27b#1 and sh-Rab27b#2 (sh-Rab27b1 + 2). Infecting with both lentiviral vectors resulted in a significant decrease of Rab27b at the mRNA and protein level, with no influence on Rab27a at the mRNA and protein level (Figures 2B–D). Cells transfected with sh-NC or sh-Rab27b1 + 2 were subsequently passaged for use in experiments.

### The Effect of Rab27b Depletion on Exosome Secretion

**FIGURE 3:**
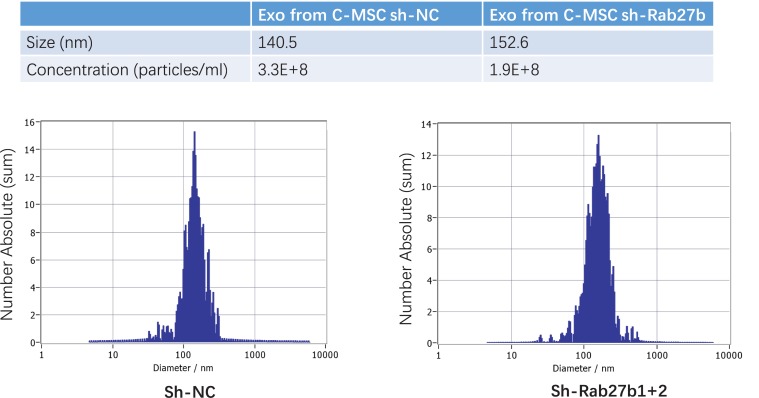
Characterization of exosomes derived from C-MSC^sh–NC^ and C-MSC^sh–Rab27b1+2^. Particle concentration and size distribution in purified particles are consistent with the size range of exosomes (average size, 140–150 nm), as measured by ZetaView^®^ Particle Tracking Analyzer.

To determine the effect of Rab27b on exosome release, we analyzed the exosomes released from C-MSCs infected with sh-NC or sh-Rab27b1 + 2. The size and concentration of pelleted structures were determined with nanoparticle tracking analysis using a ZetaView^®^ nanoparticle tracking analyzer for hydrodynamic particle size. The pellets consisted of particles with an average size of approximately 140–150 nm in diameter, consistent with the characteristic size range of exosomes. Furthermore, knockdown of Rab27b in C-MSC cells significantly reduced the concentration of exosomes released by C-MSC in culture medium (Figure 3), demonstrating that Rab27b plays a vital role in exosome secretion.

### Knockdown of Rab27b Impairs Mitochondrial Oxidative Phosphorylation in C-MSC

**FIGURE 4:**
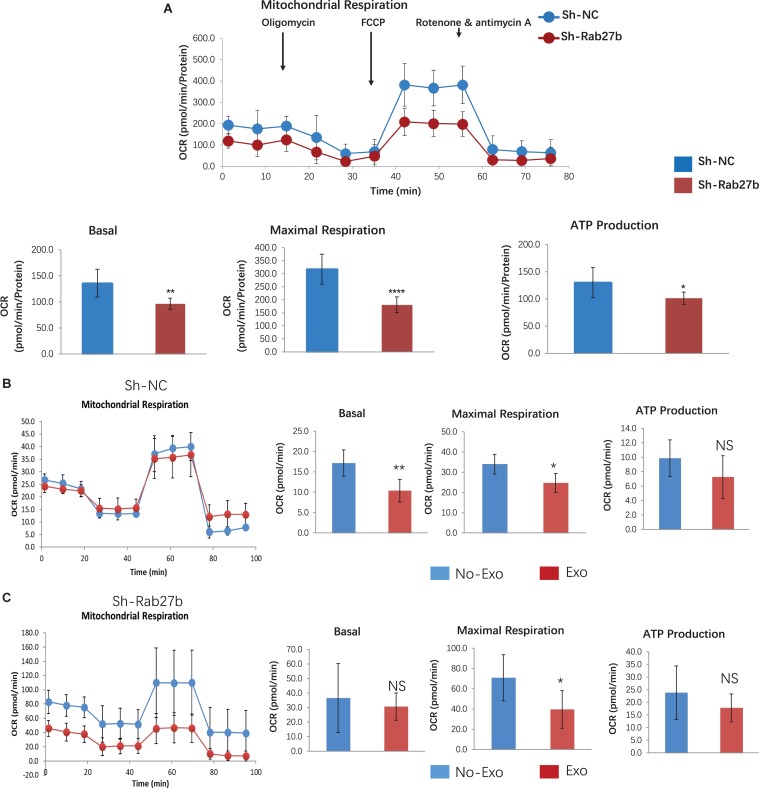
Assessment of oxygen consumption rate (OCR) in C-MSC^sh–NC^ and C-MSC^sh–Rab27b^. **(A)** Cells were exposed sequentially to oligomycin, FCCP, and rotenone/antimycin A. Vertical lines indicate time of addition of mitochondrial inhibitors. Oxygen consumption rate was measured over time using a Seahorse XFe96 Analyzer. Cell mito stress test was performed according to manufacturer’s protocol, results are normalized to total cellular protein (*n* = 7–8). **(B)** C-MSC^sh–NC^ were seeded in media with Exo-depleted FBS overnight, and primed cells with 25-μg exosomes from wild-type C-MSC for 1 h before OCR assay using a Seahorse XF24 analyzer (*n* = 5).**(C)** C-MSC^sh–Rab27b^ were seeded in media with Exo-depleted FBS overnight, and primed cells with 25-μg exosomes from wild-type C-MSC for 1 h before OCR assay using a Seahorse XF24 analyzer (*n* = 5). Results are presented as mean ± SD (*p < 0.05; **p < 0.01; ****p < 0.0001).

To determine the impact of Rab27b downregulation on cell metabolism, we quantified cellular OCR. Figure 4A shows the time course of the protocol with the injection of each compound and the impact on OCR; the results demonstrate that Rab27b deletion reduced mitochondrial oxidative phosphorylation, as evidenced by decreased basal respiration, maximal respiration, and ATP production in comparison with C-MSC^sh–NC^.

To determine the effect of wild-type C-MSC derived Exosomes on both C-MSC with/without Rab27b knockdown, we cultured both C-MSC^sh–NC^ and C-MSC^sh–Rab27b^ in media with Exo-depleted FBS and primed cells with 25 μg exosomes per well from wild-type C-MSC for 1 h before OCR assay. As shown in Figures 4B,C, exosome treatment reduced maximal respiration in both C-MSC^sh–NC^ and C-MSC^sh–Rab27b^, suggesting that external stem cell-derived exosome treatment impacts the mitochondrial respiration of C-MSC. To evaluate the FAO capacity of C-MSC^sh–NC^ and C-MSC^sh–Rab27b^, we measured OCR for beta-oxidation in C-MSC treated with BSA, FAO-BSA, FAO-BSA plus ETO. As shown in Supplementary Figure S1, both C-MSC^sh–NC^ and C-MSC^sh–Rab27b^ do not respond well to exogenous substrate (BSA-conjugated FAO); however, knockdown of Rab27b shows increased FAO-associated OCR.

**FIGURE 5:**
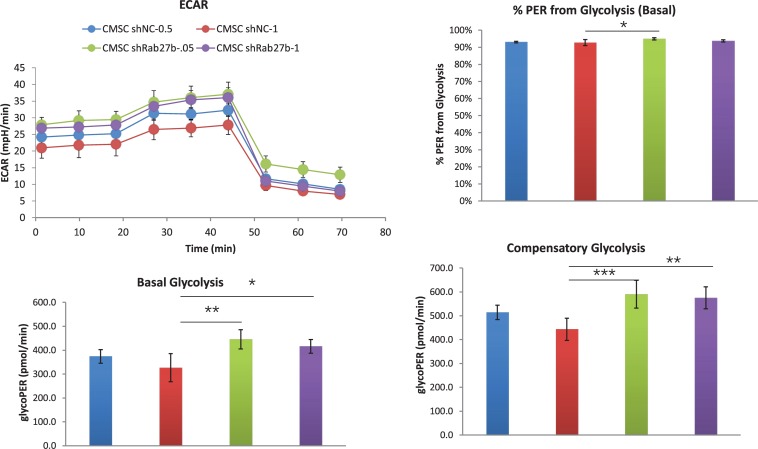
Assessment of extracellular acidification rate (ECAR) in C-MSC^sh–NC^ and C-MSC^sh–Rab27b^. Cells were exposed sequentially to 0.5 or 1 μM rotenone/antimycin A and 2-DG. Extracellular acidification rate (ECAR) was measured over time using a Seahorse XFe24 Analyzer. Measurements of basal glycolysis,% PER from glycolysis and compensatory glycolysis were calculated from the ECAR data. Results are presented as mean ± SD (*p < 0.05; **p < 0.01; ***p < 0.001, *n* = 5).

Next, we evaluated the ECAR in the absence of any substrate with further addition of glucose with the dosage of 5 and 10 mM, respectively. As shown in Supplementary Figure S2, addition of glucose in low and high dosage does not impact glycolysis and glycolytic capacity. Finally, we measured ECAR at 0.5 and 1 μM rotenone. As shown in Figure 5, knockdown of Rab27b increased basal and compensatory glycolysis at 1 μM rotenone compared to control C-MSC, suggesting higher glycolytic activity of C-MSC^sh–Rab27b^.

Taken together, these findings suggest that knockdown of Rab27b results in selectively decreased mitochondrial oxidative phosphorylation and increased glycolytic rate.

### Knockdown of Rab27b Inhibits Mitochondrial Fatty Acid β-oxidation and ETC in C-MSC

**FIGURE 6:**
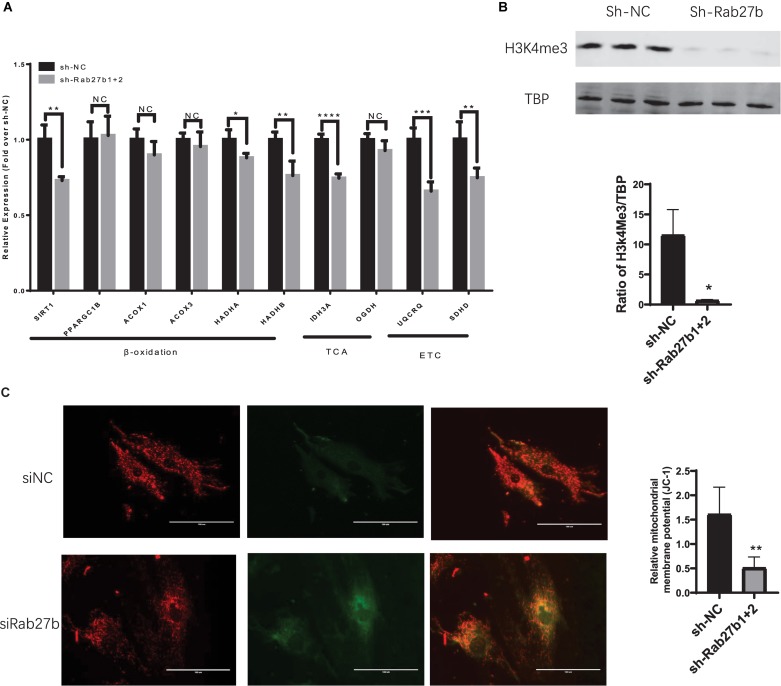
**(A)** qRT-PCR analysis of genes related to mitochondrial fatty acid oxidation; TCA, tricarboxylic acid cycle; ETC, electron transport chain. The amount of mRNA was normalized using GAPDH. (*n* = 3–4). **(B)** Western blotting of H3k4me3 protein using TBP as a loading control (*n* = 3). **(C)** Mitochondrial membrane potential was measured by JC-1 staining and the images were obtained by fluorescent microscopy. Scale bars = 100 μm. The deltapsim is expressed as the ratio of red fluorescence to green fluorescence (*n* = 5–6). Results are shown as mean ± SD (NS, *p* > 0.05, *p < 0.05, **p < 0.01, ***p < 0.001, ****p < 0.0001).

To further investigate the inhibitory effect of Rab27b knockdown on mitochondrial oxidative phosphorylation, we examined the expression of essential genes related to FAO, TCA cycle, and ETC. As shown in Figure 6, the expression of SIRT1, HADHA and HADHB (related to fatty acid β-oxidation), IDH3A (involved in TCA), UQCRQ, and SDHD (the components of the mitochondrial ETC), was decreased in C-MSC^sh–Rab27b^ in comparison to C-MSC^sh–NC^. No difference in the expression of other genes related to fatty acid β-oxidation (PPARGC1B, ACOX1, ACOX3) and TCA (OGDH) was detected between C-MSC^sh–NC^ and C-MSC^sh–Rab27b^. These data indicate that Rab27b might play a role in mitochondrial oxidative phosphorylation primarily by regulating genes that promote fatty acid β-oxidation, TCA, and ETC in C-MSCs.

To identify the mechanism of reduced expression of several genes involved in FAO, TCA, and ETC in Rab27b knockdown C-MSC, we measured the protein level of H3K4me3, a gene activation mark, in CMSC^sh–NC^ and CMSC^sh–Rab27b^. As shown in Figure 6B, knockdown of Rab27b significantly reduces the H3K4me3 level in C-MSC, suggesting that the knockdown of Rab27b can change gene expression via epigenetic regulation.

Finally, we measured the mitochondrial membrane potential between C-MSC^sh–NC^ and C-MSC^sh–Rab27b^. The mitochondrial membrane potential (deltapsim) was calculated by red/green fluorescence ratio. As shown in Figure 6C, the Rab27b knockdown decreased mitochondrial membrane potential in C-MSC.

## Discussion

This is the first observation that the small GTPase Rab27b, a regulator of exosome secretion, can affect metabolism in C-MSC. In this study, we compared cellular metabolism and energetics in adult C-MSC following knockdown of Rab27b. Knockdown of the Rab27b gene increased cell glycolysis, but significantly inhibited oxidative phosphorylation in C-MSCs and selectively down-regulated the expression of the key genes involved in fatty acid β-oxidation, TCA and ETC, indicating that Rab27b plays a regulatory role in C-MSCs metabolism.

Under normal conditions, about 60–90% of ATP required for the continuous contractile activity of the heart is primarily produced by oxidation of fatty acids (FA), while the rest is derived from the oxidation of pyruvate (van der Vusse et al., 1992). Fatty acids are completely oxidized to water and carbon dioxide through β-oxidation, the TCA cycle, and the ETC, producing ATP (Wanders et al., 2010). At the same time, TCA cycle and ETC are also involved in the oxidative metabolism of carbohydrates and proteins (Akram, 2014). Flaherty et al. (2019) reported that fat cells can regulate fat metabolism by direct release of neutral lipids from exosomes. Ying et al. (2017) demonstrated that exosome microRNAs from adipose tissue macrophages were shown to regulate systemic glucose metabolism by regulating fat cell function.

Furthermore, a recent study suggested that exosome secretion can attenuate cellular stress and maintain cellular homeostasis by exporting various unnecessary or harmful materials (Takahashi et al., 2017). Thus, in addition to participating in paracrine signaling, exosome formation and secretion may play a fundamental role in regulating intracellular functions. However, Rab27b, which regulates exosome secretion (Ostrowski et al., 2010), has not been demonstrated to modulate cellular metabolism. In this study, we first confirmed that knockdown of Rab27b decreased exosome secretion in C-MSCs. Next, we found that knockdown of Rab27b in C-MSC results in a significant reduction in mitochondrial oxidative metabolism, suggesting that Rab27b might be important to maintain fatty acid oxidative metabolism in C-MSC.

To investigate the mechanism by which knockdown of Rab27b inhibits mitochondrial oxidative metabolism in C-MSCs, we analyzed the expression of β-oxidation, TCA, and ETC-related genes in C-MSC^sh–NC^ and C-MSC^sh–Rab27b^. We observed that knockdown of Rab27b not only reduced the expression of SIRT1, HADHA, and HADHB related to β-oxidation, IDH3A related to TCA, but also decreased the expression of UQCRQ and SDHD involved in ETC. SIRT1 is a highly conserved member of the family of NAD + -dependent Sir2 histone deacetylases (Rodgers et al., 2005) and has also been reported to sense the redox shifts and integrate mitochondrial metabolism through post-transcriptional regulation of the transcription factors and histones (Vachharajani et al., 2016). HADHA and HADHB, respectively, encode the α and β subunits of the mitochondrial trifunctional protein, catalyzing the last three steps of mitochondrial beta-oxidation of long-chain fatty acids (Diebold et al., 2019; Ljubkovic et al., 2019) also observed that in diabetic myocardium, a decrease in HADHA reduces the mitochondrial β-oxidation capacity and increases intracellular lipid accumulation, resulting in a negative impact on the heart. Moreover, Vasilescu et al. (2018) demonstrated that HADHB is directly associated with severe childhood-onset cardiomyopathies.

IDH3A encodes the alpha subunit of IDH3, which is one of three isozymes of NAD (+)-dependent isocitrate dehydrogenase that localize in the mitochondrial matrix and is an allosteric regulatory rate-limiting step that catalyzes the TCA cycle. It is necessary for the TCA cycle to progress and generate NADH, which feeds into oxidative phosphorylation to generate ATP (Findlay et al., 2018). SDHD encodes a member of complex II of the respiratory chain, which is responsible for the oxidation of succinate and represents an intersection between the mitochondrial respiratory chain for which an important function in cardiopulmonary oxygen sensing has been demonstrated, and the Krebs cycle, a central element of α-KA metabolism (Muhling et al., 2010). UQCRQ encodes a subunit of ubiquinol-cytochrome c reductase complex III, which is a part of the mitochondrial respiratory chain (Wen and Garg, 2004). Furthermore, cytochrome C reductase complex III has been identified as a major producer of superoxide and derived reactive oxygen species (ROS) in the mitochondrial respiratory chain (Drose and Brandt, 2012; Bleier and Drose, 2013). Mitochondrial ROS can lead to oxidative damage, cell death and abnormal immune responses in the heart after ischemia–reperfusion injury (Chouchani et al., 2014). Therefore, UQCRQ might also be involved in mitochondrial ROS production and myocardial dysfunction (Lu et al., 2019).

Our study shows that knockdown of Rab27b significantly reduces the level of H3K4me3, a gene activation marker, in C-MSC^sh–Rab27b^ compared to C-MSC^sh–NC^, suggesting that the knockdown of Rab27b can change gene expression via epigenetic regulation. Vizoso et al. (2015) recently reported that the epigenetically reactivated TBC1D16-47KD transcript is able to reduce EGFR activity in metastatic cells through members of Rab family. We will identify the mechanism how Rab27b modifies histone H3 methylation in our future study.

## Conclusion

Our findings highlight, for the first time, a novel role for Rab27b in maintaining oxidation of fatty acids in C-MSCs, possibly by regulating the expression of key genes involved in fatty acid β-oxidation, TCA, and ETC. Future studies will be needed to determine the underlying epigenetic and gene specific mechanism whereby Rab27b controls metabolism and regulates oxidation of fatty acids in C-MSCs.

## Data Availability Statement

All datasets generated for this study are included in the article/Supplementary Material.

## Ethics Statement

Animal treatment protocols were approved by, and conducted in accordance with, animal welfare regulations of the Institutional Animal Care and Use Committee of the Medical College of Georgia.

## Author Contributions

YT: conceptualization. YJ: writing. YJ, XS, YS, JC, and YL: experiments. NW and YT: writing-review and editing.

## Conflict of Interest

The authors declare that the research was conducted in the absence of any commercial or financial relationships that could be construed as a potential conflict of interest.
