# Supplementary material for: The Small GTPases Rab27b Regulates Mitochondrial Fatty Acid Oxidative Metabolism of Cardiac Mesenchymal Stem Cells
Source: Front Cell Dev Biol. 2020 Apr 15;8:209. doi: 10.3389/fcell.2020.00209 (PMC7174509; doi:10.3389/fcell.2020.00209)
Supplement: Supplementary file 1 [file Presentation_1.pdf]

Suppl. Figure 1

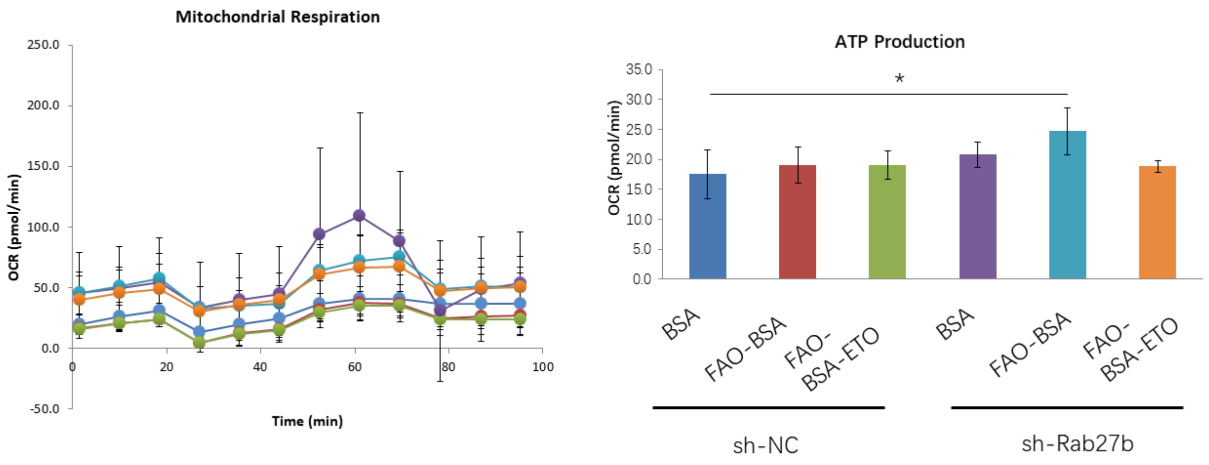

Suppl. Figure 1. Assessment of oxygen consumption rate (OCR) in C-MSC$^{sh\text{-}NC}$ and C-MSC$^{sh\text{-}Rab27b}$. **(A)** Cells were cultured in substrate-limited medium overnight, and exposed sequentially to FAO medium, etomoxir (40µM), and Palmitate:BSA or BSA before OCR assay. Oxygen consumption rate (OCR) was measured over time using a Seahorse XFe24 Analyzer. Cell mito stress test was performed according to manufacturer's protocol. Measurements of ATP production were calculated from the OCR data. Results are presented as mean ± SD (*, $P<0.05$, n=3-4).

Suppl. Figure 2

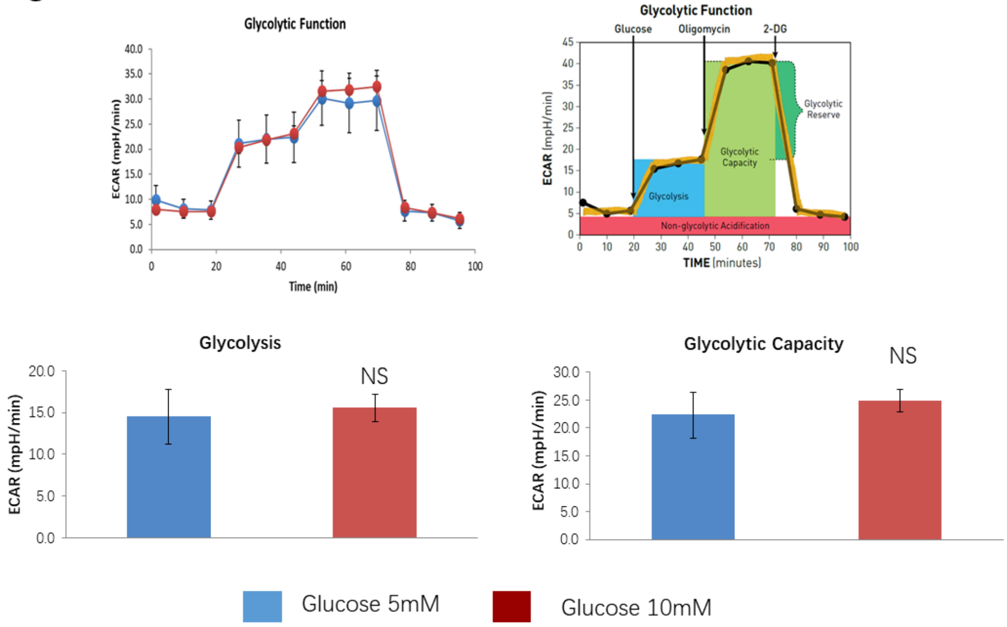

Suppl. Figure 2. Assessment of extracellular acidification rate (ECAR) in C-MSC. Cells were subjected to absence of glucose and sequentially to 5mM or 10mM glucose, rotenone/antimycin A and 2-DG. Extracellular acidification rate (ECAR) was measured over time using a Seahorse XFe24 Analyzer. Measurements of basal glycolysis, and glycolytic capacity were calculated from the ECAR data. Results are presented as mean ± SD (NS, $p>0.05$, $n=5$).
